# Molecular Diffusivity of Click Reaction Components: The Diffusion Enhancement Question

**DOI:** 10.1021/jacs.1c11754

**Published:** 2022-01-14

**Authors:** Nasrollah Rezaei-Ghaleh, Jaime Agudo-Canalejo, Christian Griesinger, Ramin Golestanian

**Affiliations:** † Department of NMR-Based Structural Biology, Max Planck Institute for Biophysical Chemistry, Am Faßberg 11, D-37077 Göttingen, Germany; # Institut für Physikalische Biologie, Heinrich-Heine-Universität Düsseldorf, Universitätsstraße 1, D-40225 Düsseldorf, Germany; ‡ Department of Living Matter Physics, Max Planck Institute for Dynamics and Self-Organization, Am Faßberg 17, D-37077 Göttingen, Germany; ∥ Rudolf Peierls Centre for Theoretical Physics, University of Oxford, Oxford OX1 3PU, United Kingdom

## Abstract

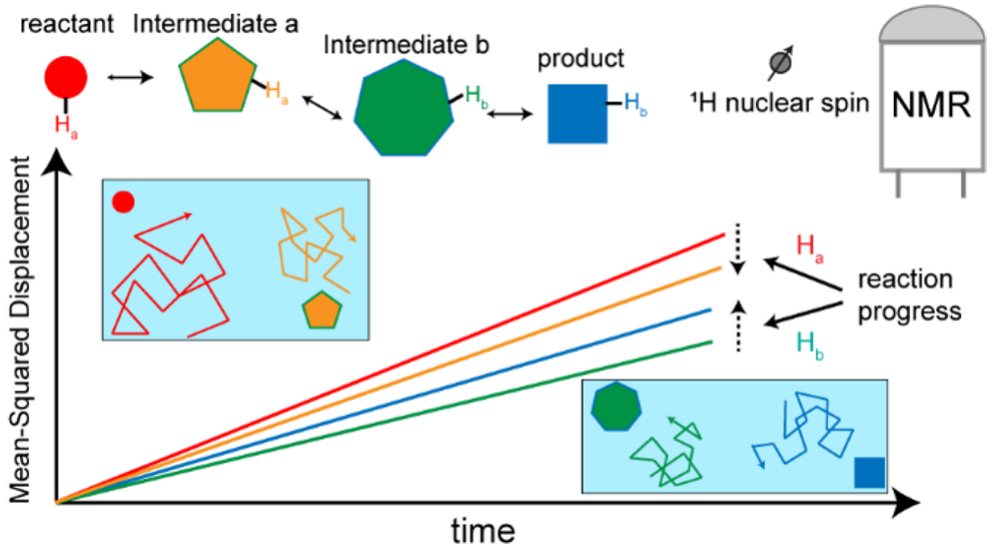

Micrometer-sized objects are widely known to exhibit chemically driven motility in systems away from equilibrium. Experimental observation of reaction-induced motility or enhancement in diffusivity at the much shorter length scale of small molecules is, however, still a matter of debate. Here, we investigate the molecular diffusivity of reactants, catalyst, and product of a model reaction, the copper-catalyzed azide–alkyne cycloaddition click reaction, and develop new NMR diffusion approaches that allow the probing of reaction-induced diffusion enhancement in nanosized molecular systems with higher accuracy than the state of the art. Following two different approaches that enable the accounting of time-dependent concentration changes during NMR experiments, we closely monitored the diffusion coefficient of reaction components during the reaction. The reaction components showed distinct changes in the diffusivity: while the two reactants underwent a time-dependent decrease in their diffusivity, the diffusion coefficient of the product gradually increased and the catalyst showed only slight diffusion enhancement within the range expected for reaction-induced sample heating. The decrease in diffusion coefficient of the alkyne, one of the two reactants of click reaction, was not reproduced during its copper coordination when the second reactant, azide, was absent. Our results do not support the catalysis-induced diffusion enhancement of the components of the click reaction and, instead, point to the role of a relatively large intermediate species within the reaction cycle with diffusivity lower than that of both the reactants and product molecule.

## Introduction

Molecular machines which convert chemical energy into kinetic energy or mechanical work are key players in natural and synthetic biology and nanotechnology.^1−3^ Of particular interest to applications such as drug delivery and nanorobotics is the transduction of chemical energy into translational motion in the bulk of a fluid.^4,5^ Artificial microscopic particles such as bimetallic rods, Janus particles, and enzyme-coated beads are known to undergo self-propelled directed motion powered by their surface catalytic activity, with mechanisms that are by now well understood.^6,7^ Over long time scales, this ballistic motion is randomized by rotational diffusion, leading to greatly enhanced diffusive behavior.^8,9^ There have also been theoretical proposals for achieving stochastic swimming at the nanoscale by breaking the detailed balance, akin to how biological molecular motors function.^10,11^ More recently, experiments have reported that also single enzymes may experience catalysis-induced enhanced diffusion,^12−14^ although the possible underlying mechanisms and even the existence of this phenomenon are still under debate.^15−19^ Continuing the quest toward translational motion at increasingly smaller scales, it was later claimed that even molecular-scale systems (a Grubbs catalyst) exhibit enhanced diffusion during catalysis,^20,21^ although this was subsequently shown to be due to a convection artifact in the measurements.^22^

A recent report, however, has reinvigorated the idea of enhanced diffusion during molecular catalysis.^23^ In these experiments, it was claimed that the mobility of reactant molecules in a family of organic chemical reactions, including the Cu(I)-catalyzed azide–alkyne cycloaddition (CuAAC) reaction, are boosted during catalysis.^23^ With the word “boosted”, it is implied that the underlying mechanism is an active, propulsive one, akin to stochastic swimming^10,11^ associated with the (free) energy released during each catalytic event. From a theoretical perspective, however, propulsive motion can only be observed over a very short period of time, as the negligible inertial effects will lead to rapid dissipation of the kinetic energy from sudden “kicks” into the environment, leading to a randomization of the propulsion by rapid rotational diffusion.^15,17−19,24^ In addition to theoretical concerns, this report has been hotly debated from a technical perspective, especially with regard to the challenges of diffusion measurement by NMR.^25−31^

Pulse field gradient (PFG) NMR is the only technique that can enable the monitoring of molecular diffusivity at atomic scale. Here, molecular “diffusivity” is the rate of (mass) diffusion under a concentration gradient, as defined through Fick’s equations of diffusion, and is equivalent to “diffusion coefficient” at equilibrium, as implicated by the fluctuation–dissipation theorem. The technique allows us to determine the diffusion coefficients for the various molecular components of a reaction mixture, including reactants, catalysts, intermediate species, products, and solvent molecules. The PFG-NMR technique relies on spatial encoding of molecules via application of a magnetic field gradient along the *z*-axis of an NMR tube, through which the frequency of nuclear spins sitting on the molecules would carry *z*-coordinate information.^32^ After a diffusion delay, during which the molecules undergo diffusion in different directions, including the *z*-axis, the spatial information is decoded through application of another field gradient pulse with the same magnitude but the opposite sign. The NMR signals of nondiffusing molecules would therefore be completely recovered after the second gradient pulse and its consequent reversing of nuclear spin frequencies, while the diffusing molecules will undergo NMR signal attenuation dependent on their displacement along the *z*-axis and the strength of the magnetic field gradient. The NMR signal intensity versus gradient field strength data will then allow determining diffusion coefficients separately for each NMR-resolved signal and its underlying molecular species. Furthermore, when a chemical reaction takes place in the NMR sample, the real-time PFG-NMR experiments enable monitoring of molecular diffusion over the course of the reaction. However, in such cases, proper technical adjustments in NMR diffusion experiments and data analysis should be made to account for NMR signal intensity changes due to the kinetics of the reaction and consequent changes in the concentration, NMR relaxation properties, etc.^33^

Here, we investigate molecular diffusion during the CuAAC reaction using the adjusted NMR diffusion experiments and introduce two ways in which artifacts due to time-dependent signal intensities in NMR diffusion experiments can be identified and corrected, hence enabling detection and quantification of potential reaction-induced diffusion enhancements in nanosized molecular systems. It is demonstrated that the two reactants, catalyst, and product of this reaction experience distinct reaction-dependent alterations in their diffusivity. Our results do not support the uniform catalysis-induced diffusion enhancement in CuAAC reaction, as suggested in ref (23), but instead point to the role of intermediate species in the CuAAC reaction cycle as the primary cause of reaction-dependent molecular mobility alterations.

**Scheme 1:**
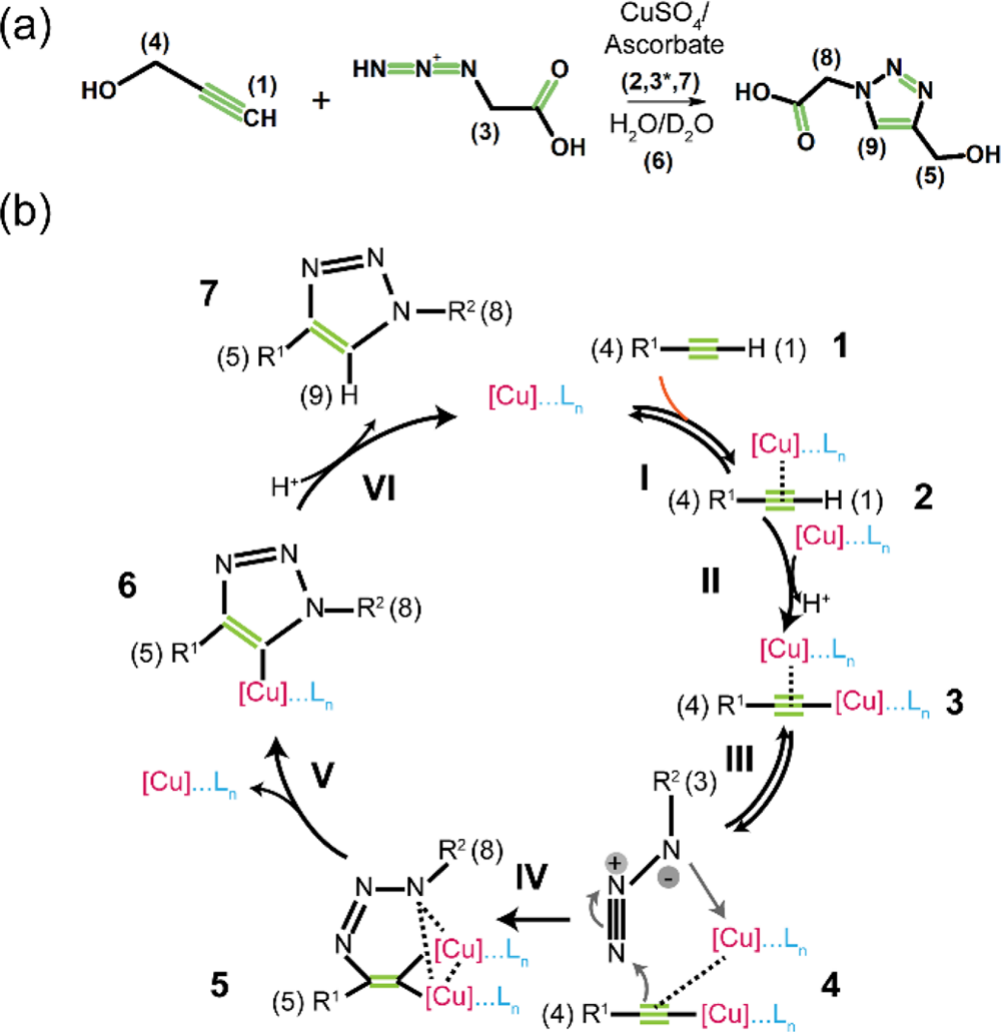
(a) Copper-Catalyzed Azide–Alkyne Cycloaddition Click Reaction, with Numbers (1)–(9) Corresponding to the NMR Signals Studied Here, and (b) Schematic Depiction of the Catalytic Cycle, Proceeding through Steps I–VI, Involving Chemical Species **1**–**7**

The CuAAC reaction is one of the primary examples of click reactions which transforms organic azides and terminal alkynes into the corresponding 1,2,3-triazoles (Scheme 1a).^34,35^ Since Cu(I) is the least thermodynamically stable oxidation state of copper, a combination of Cu(II) salt and ascorbate, a mild reducing agent, is often used as a source of Cu(I) in CuAAC reactions performed in aqueous solutions.^35^ Unlike the uncatalyzed reaction, which requires much higher temperatures and produces mixtures of 1,4- and 1,5-disubstituted triazole regioisomers, the copper-catalyzed reaction is fast at room temperature and produces nearly pure 1,4-disubstituted triazoles. This is because catalysis by copper converts the mechanism of the cycloaddition reaction into a sequence of discrete steps, where the activation energy barrier for the key rate-determining C–N bond formation is reduced compared to that in the uncatalyzed reaction.^36,37^ Much remains to be understood about the complex mechanism of the CuAAC reaction, but several lines of experimental evidence and DFT calculations propose that the reaction begins with the recruitment of a π-bound copper ion to the alkyne molecule, which acidifies its terminal proton and therefore facilitates its replacement with a second copper ion, hence forming of a σ-bond copper acetylide (Scheme 1b, steps I and II). Then, the reversible coordination of the dinuclear copper intermediate with the azide molecule leads to the synergistic nucleophilic activation of the alkyne and electrophilic activation of the azide and drives the formation of the first C–N bond within a strained copper metallacycle (Scheme 1b, steps III and IV). The subsequent energetically favorable steps of copper triazolide formation (step V) and copper substitution by proton (step VI) will then culminate in the formation of the triazole molecule as the reaction product.^36−41^ Notably, the unique catalytic activity of a copper ion is rooted in its combined propensity of engaging in π- and σ-interactions with terminal alkynes *and* rapid exchange of these and other ligand molecules (including solvent molecules), especially in aqueous solutions, in its coordination sphere.^36^

## Results and Discussion

**Figure 1:**
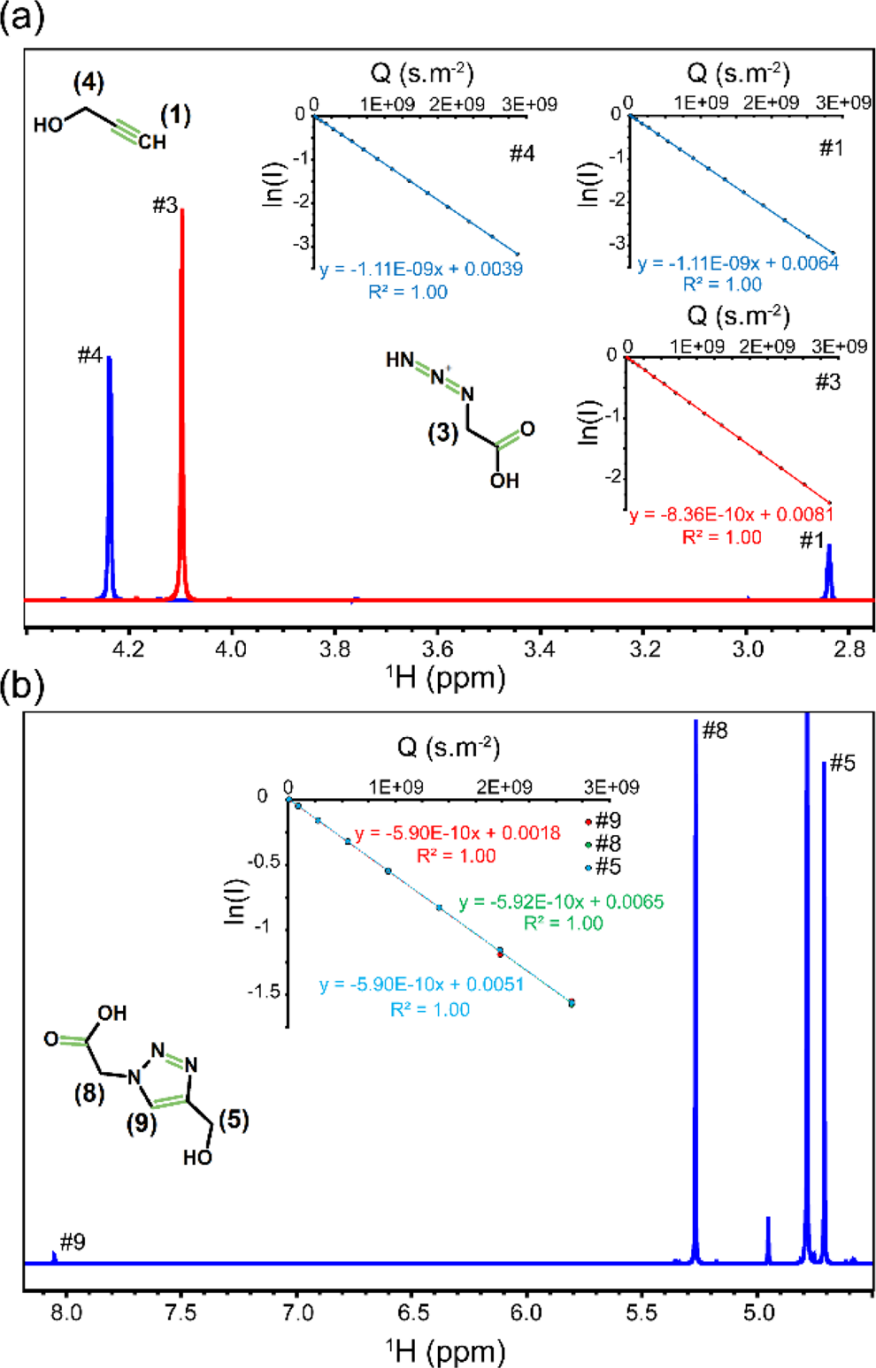
1D ^1^H NMR spectra of the two reactants (a: alkyne, blue; azide, red) and single product (b: triazole) of the click reaction measured in isolation (for reactants) or after completion of reaction (for product). The NMR signals are assigned according to the 2D chemical structures shown. The gradient-dependent NMR intensity attenuations of different NMR signals in log-quadratic scale are shown as insets, in which the linear slopes represent diffusion coefficients of reactants and product molecules (*D*_0_) used as reference in this study.

The 1D ^1^H NMR spectrum of the sample containing 0.2 M prop-2-ynol (henceforth, alkyne or reactant 1) in D_2_O is shown in Figure 1a. The two resonances of the alkyne molecule were observed at 4.238 and 2.838 ppm, respectively corresponding to the two methylene protons (−CH_2_, signal #4) and one terminal proton (≡CH, signal #1). Then, the diffusion coefficient of the alkyne molecule was measured via the PFG-NMR method. In general, the excellent linearity of the NMR signal intensity vs gradient field strength in log-quadratic scale, as illustrated in the insets of Figure 1 and Figure S1 in the Supporting Information (SI)), allowed precise determination of the diffusion coefficient of alkyne (and other) molecules. In addition, the convection-compensated PFG-NMR experiments confirmed that the contribution of convection to molecular mobility was negligible (see the Experimental Section in the SI).^42^ The NMR diffusion measurement yielded the same diffusion coefficient (*D*_0_, where the subscript 0 denotes diffusion coefficient at equilibrium, i.e., in the absence of chemical reaction) of 11.1 × 10^–10^ m^2^·s^–1^ for signals #1 and #4, as expected for them sitting on the same diffusing molecule.

Figure 1a, shows the 1D ^1^H NMR spectrum of the sample containing 0.2 M 2-azidoacetic acid (henceforth, azide or reactant 2) in D_2_O. The resonance corresponding to the two methylene protons (−CH_2_, signal #3) of the azide molecule was observed at 4.097 ppm, and the associated *D*_0_ was 8.4 × 10^–10^ m^2^·s^–1^. The absolute *D*_0_ values of alkyne and azide molecules obtained here were slightly larger than the previously reported values (ca. 7 and 9%, respectively),^28^ which is probably caused by small differences in temperature, imperfect compensation for convection artifacts, and/or gradient calibration errors; however, the ratio between *D*_0_ of alkyne and azide molecules was 1.32, in close agreement with the previously obtained value of 1.35.^28^ The sample containing alkyne and azide molecules each at 0.2 M concentration in the absence of any catalyst did not show any considerable change in the chemical shifts of the three signals belonging to the two reactants or their associated diffusion coefficients. In addition, the NMR spectrum of the mixed sample did not change during an overnight incubation at 298 K, indicating that the rate of uncatalyzed click reaction was negligible at this temperature.

The 1D ^1^H NMR spectrum of the sample containing 64 mM sodium ascorbate (henceforth, ascorbate or catalyst) in D_2_O is shown in SI, Figure S1a, where three signals were observed, at 4.529, 4.036, and 3.762 ppm, respectively corresponding to the methine proton (−CH) of the ring (signal #7) and the side chain (signal #3*) and the two methylene protons (−CH_2_, signal #2) of the side chain of ascorbate molecules. The hydroxyl (−OH) protons are not expected to appear as separate resonances, as they are in rapid exchange with the solvent at this pH. The same *D*_0_ of 5.8 × 10^–10^ m^2^·s^–1^ was observed for all three signals (Figure S1a, inset). Addition of 16 mM CuSO_4_ led to a slight downfield displacement of peaks #2 and #3* and alterations in their line width and splitting pattern, while peak #7 disappeared and four new peaks at chemical shifts of 4.707, 4.623, 4.308, and 4.202 ppm emerged (Figure S1b). The peak at 4.707 ppm underwent gradual downfield displacement, indicating a chemical reaction occurring on the time scale of minutes (Figure S1b, inset). The newly emerged peaks belonged to the oxidation products of ascorbate, such as dehydroascorbic acid, induced in the presence of Cu(II) ions. After completion of reaction, signals #2 and #3* exhibited the same diffusion coefficient of 5.9 × 10^–10^ m^2^·s^–1^, very close to the *D*_0_ value obtained for the ascorbate molecule before addition of CuSO_4_. The newly emerged signals, however, showed slightly larger diffusion coefficients in the range of (6.0–6.4) × 10^–10^ m^2^·s^–1^, consistent with them belonging to different molecular species generated through oxidation of ascorbate to smaller molecules.

**Figure 2:**
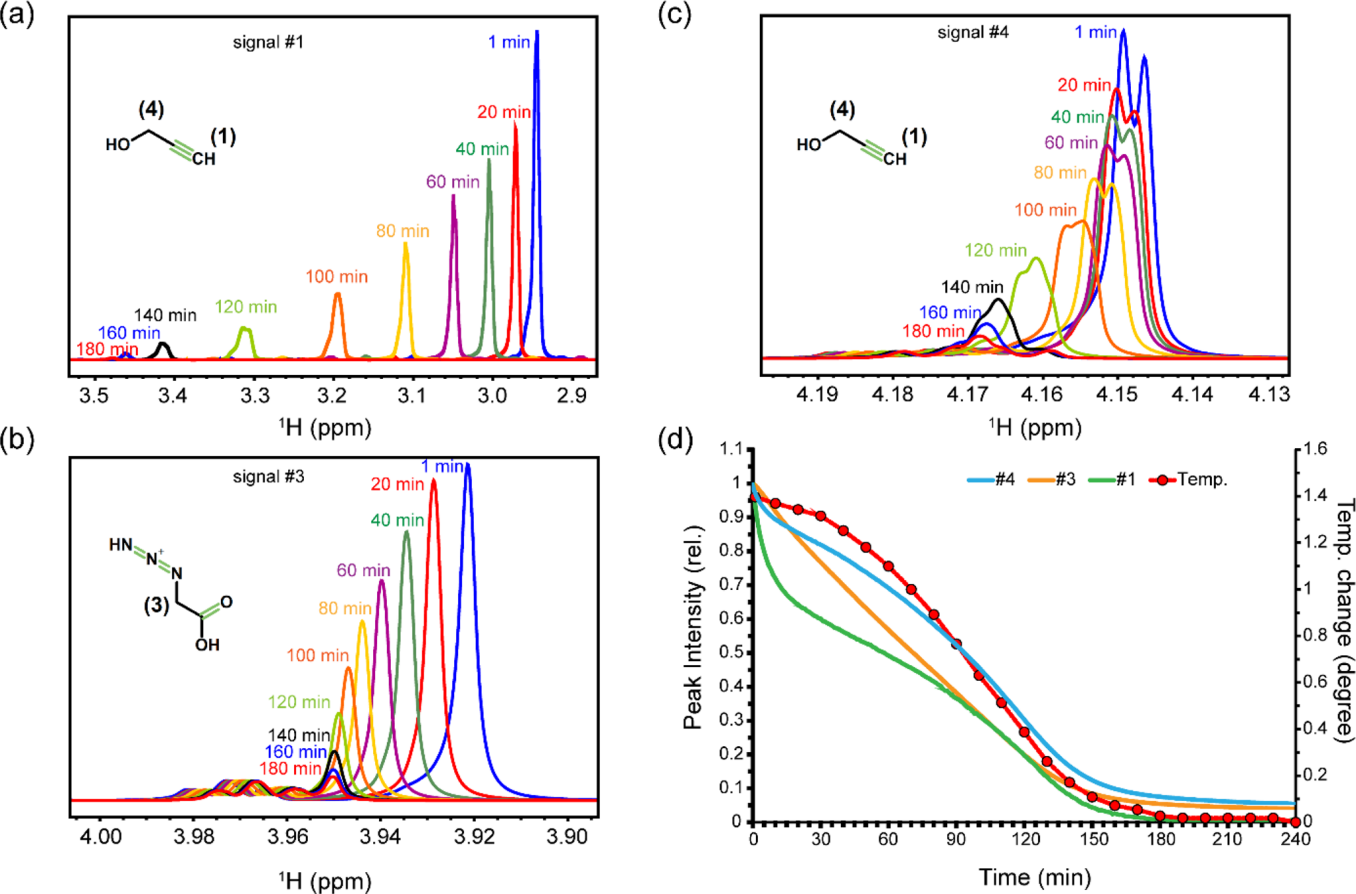
Kinetics of reactant consumption during click reaction monitored through real-time 1D ^1^H NMR spectra. Time-dependent changes in signals #1 (terminal proton of alkyne), #3 (methylene protons of azide), and #4 (methylene protons of alkyne) are shown in panels (a)–(c), along with 2D chemical structures of the reactant molecules. Time-dependent changes in NMR signal intensities are shown in (d). Note the difference in the kinetic profiles of the three signals, reflecting their different entry points to the click reaction catalytic cycle. In (d), time-dependent changes in the temperature of the NMR sample during the click reaction are shown with respect to the equilibrium temperature.

Next, to obtain the diffusion coefficient of the 1,4-disubstituted 1,2,3-triazole molecule produced by the CuAAC reaction (henceforth, triazole or product), we started the reaction by adding 16 mM CuSO_4_ and 64 mM sodium ascorbate to the mixture of alkyne and azide molecules, each at 200 mM concentration, and let the catalyzed reaction proceed to completion during overnight incubation at 298 K. The 1D ^1^H NMR spectrum of the sample after reaction completion shows three signals at 8.050 (signal #9), 5.268 (signal #8), and 4.709 ppm (signal #5) belonging to the product triazole molecule (Figure 1b). The diffusion coefficients associated with the three product signals were all approximately 5.9 × 10^–10^ m^2^·s^–1^. Overall, the diffusion coefficients of the four molecules studied here followed the order *D*_alkyne_ (1.1 × 10^–9^) > *D*_azide_ (0.84 × 10^–9^) > *D*_triazole_ (0.59 × 10^–9^) ≈ *D*_ascorbate_ (0.58 × 10^–9^), in qualitative agreement with the molecular-mass-based estimation of their diffusion coefficients (their molecular masses are 56.06, 101.06, 157.13, and 176.12 Da, respectively). Next, we monitored through real-time 1D ^1^H NMR experiments how the three proton signals of the alkyne and azide reactant molecules varied over the course of the CuAAC reaction triggered by addition of CuSO_4_ and sodium ascorbate. As shown in Figure 2a, signal #1, belonging to the terminal proton of the alkyne molecule, underwent a time-dependent chemical shift displacement downfield, along with a drastic decrease in signal intensity. These changes are induced by π-coordination of alkyne to copper ion, which leads to acidification of the terminal alkyne proton and increases the exchange rate with deuterium ions in solvent.^31^

**Figure 3:**
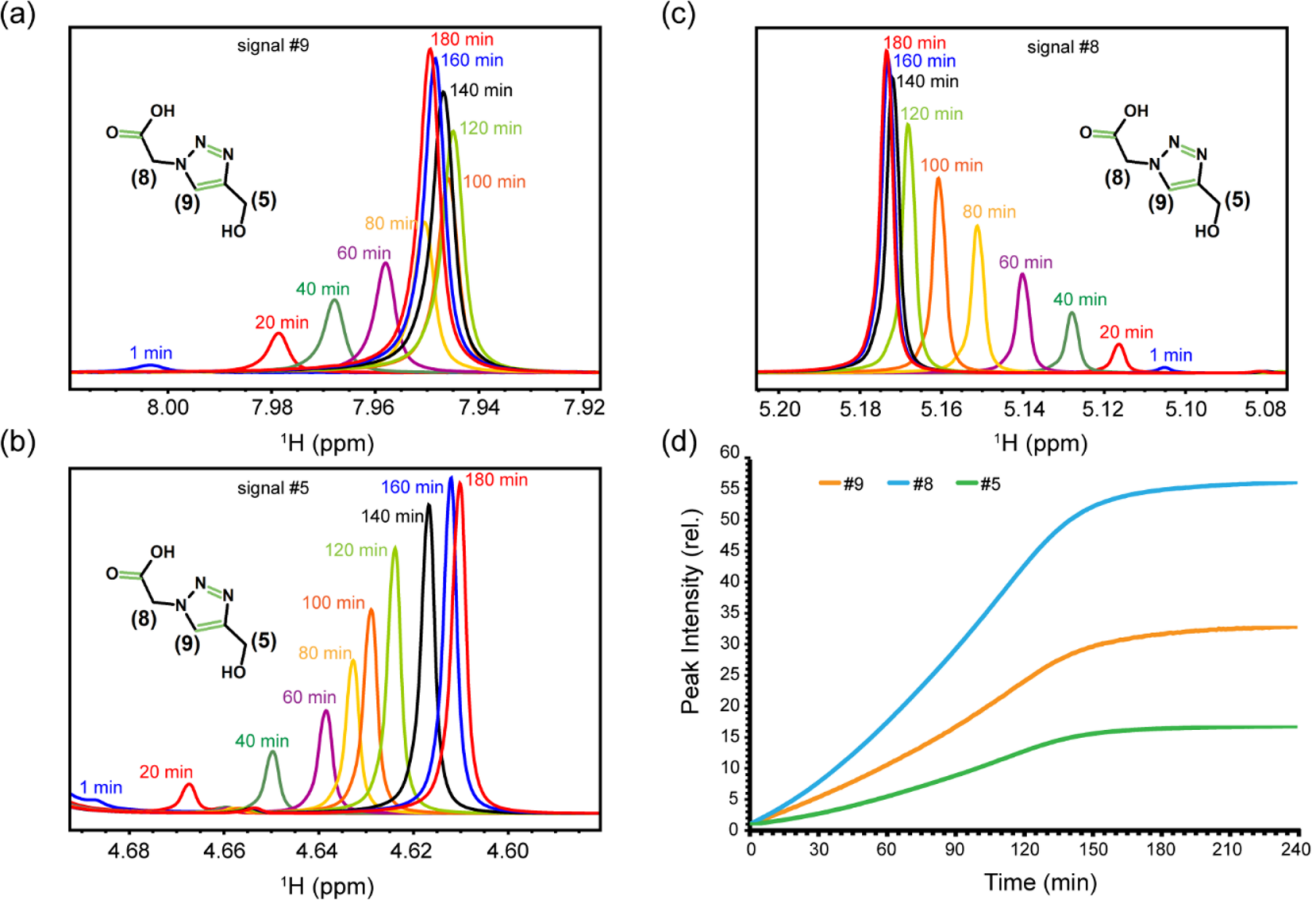
Kinetics of product formation during the click reaction monitored through real-time 1D ^1^H NMR spectra. Time-dependent changes in signals #5, #8, and #9, belonging to the product molecule (triazole), are shown in panels (a)–(c), along with its 2D chemical structure. In (a), note that the direction of chemical shift displacement is reversed after ca. 120 min of reaction. Time-dependent changes in NMR signal intensities are shown in (d).

Signals #4 and #3, belonging to the methylene protons of the alkyne and azide molecules, respectively, experienced similar albeit smaller downfield chemical shift displacement, as well as intensity loss (Figure 2b–d). On the other hand, signals #5, #8, and #9, belonging to the product molecule, showed time-dependent intensity gains, which in the case of signals #5 and #9 was accompanied by upfield chemical shift displacements, while a downfield chemical shift displacement was observed for signal #8 (Figure 3a–d). Interestingly, the upfield chemical shift displacement of signal #9 was partially reversed before the completion of reaction, suggesting the presence of multiple, i.e., more than two, chemical species underlying this signal. In general, the presence of single NMR signals per proton species of the reactant and product molecules indicates that the related exchange processes along the reversible steps of the reaction cycle are fast with respect to the relevant NMR chemical shift time scales. In addition, the NMR evidence for the presence of multiple species underlying signal #9 indicates that the reaction mechanism is more complex than what is shown in Scheme 1b.

Subsequently, we monitored how the temperature of the NMR sample changes along the CuAAC reaction. As estimated through the chemical shift difference between the reference tetramethylsilane (TMS) and residual water (HDO) proton signals and its temperature dependence,^43^ the (average) temperature of the NMR sample was higher by around 1.4 °C in the beginning of the click reaction and gradually decreased during the first steps of reaction cycle, as shown previously,^36−38^ which implies that the heat generated within the NMR tube could not be dissipated instantaneously. Based on the temperature dependence of water viscosity, the average rise of 1.4 °C in the temperature reduces the sample viscosity by 3.1% and is therefore expected to increase the diffusion coefficients by ca. 3.6%.

Next, we employed real-time PFG-NMR diffusion measurements in order to monitor how the CuAAC reaction influences the molecular mobility of reactants, product, and catalyst molecules. Since the NMR signals of reactants and product molecules underwent considerable intensity changes during NMR diffusion measurements, as illustrated in Figures 2 and 3, we needed to account for the reaction-time-dependent signal intensity changes in addition to the gradient-field-dependent intensity changes. Otherwise, it would lead to a systematic over- or under-estimation of diffusion coefficients derived through the standard Stejskal–Tanner (ST) eq (SI, eq S1), depending on the decreasing or increasing trend of signal intensities, respectively.^33^ To avoid such artifacts, we employed an approach in which, unlike the standard PFG-NMR diffusion experiment in which the gradients are ordered in increasing strength, the order of gradient strengths was shuffled in a way that the correlation between reaction time (and its consequent signal intensity changes) and gradient strength approached zero. This approach eliminates the systematic error in diffusion coefficients due to kinetic effects, although it may enhance the scattering of intensity vs gradient field strength data and therefore increase the random error. To reduce the potential random error caused by gradient shuffling, we utilized the scan-interleaved NMR experimental scheme so that the time interval between two consecutive gradient fields was decreased by a factor of 8 or 16 (depending on the number of scans used in NMR diffusion experiments).

**Figure 4:**
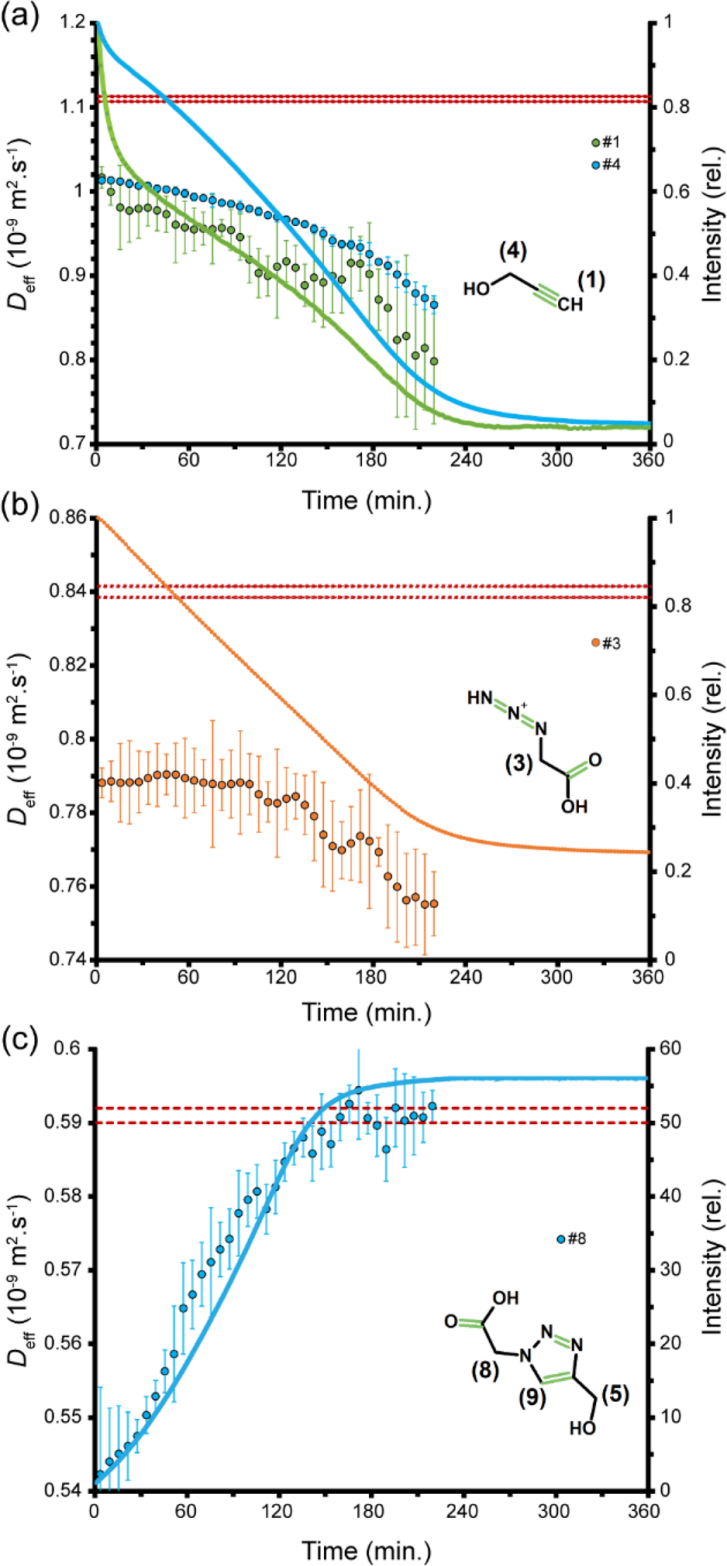
Diffusion of the reactants and product molecules during the click reaction monitored through real-time PFG-NMR experiments. Both the alkyne (a) and azide (b) molecules begin with an effective diffusion coefficient (*D*_eff_) smaller than their reference diffusion coefficients (*D*_0_, average ± std dev, shown as dashed lines) and exhibit further decay during the reaction. The product molecule triazole (c), however, shows a time-dependent rise in *D*_eff_. The time-dependent changes in signal intensity are shown as lines.

First, we monitored how the effective diffusion coefficient (*D*_eff_) of reactants **1** (alkyne) and **2** (azide) changed over the course of the CuAAC reaction. Both of the signals belonging to the alkyne molecule, i.e., signals #1 and #4, started with a *D*_eff_ around 8% lower than the reference *D*_0_ of 11.1 × 10^–10^ m^2^·s^–1^ measured in the absence of reaction (Figure 4a). Along with the progress of the reaction, the two signals exhibited a further time-dependent drop in their *D*_eff_ values, albeit with different patterns: the *D*_eff_ associated with signal #1, i.e., the terminal alkyne proton, underwent two phases of rapid decay intervened by a phase of relative stability, while signal #4 exhibited an initial slow decrease in *D*_eff_ followed by a more rapid drop. The time-dependent decay in mobility is in line with the formation of Cu-alkyne and 2Cu-alkyne complexes (species **2** and **3**, respectively, in Scheme 1b), which are significantly larger than the uncomplexed alkyne molecule (species **1**), especially when we consider the (dynamic) network of ligand (e.g., water) molecules in the coordination sphere of copper ions.

Furthermore, as suggested by the reaction mechanism depicted in Scheme 1b, the *D*_eff_ of signal #1 represents the population-weighted average of the diffusion coefficients of species **1** and **2**, while the *D*_eff_ of signal #4 is the corresponding average for species **1**, **2**, and **3**. Consequently, it is not surprising that the two signals of the alkyne molecule followed distinct time-dependent changes in their *D*_eff_ during the reaction. Similar to the signals of alkyne molecules, the azide signal #3 started with a *D*_eff_ around 6% lower than the reference *D*_0_ of 8.4 × 10^–10^ m^2^·s^–1^. However, in line with the later entry of azide to the reaction cycle, its *D*_eff_ remained nearly constant during the first 60–90 min of reaction (Figure 4b). It was then followed by a clear time-dependent decay in mobility, as expected for reversible coordination of azide with the 2Cu-alkyne complex (species **4**). The initial drop in *D*_eff_ of azide is likely due to its known, albeit weak, coordination with copper ions.^36^

Next, the *D*_eff_ of the product triazole molecule was probed through its well-resolved signals #8 and #9 (Figure 4c and SI, Figure S2). Interestingly, and in contrast with the two reactants, the triazole molecule exhibited a clear time-dependent increase in *D*_eff_, so that the limiting value of *D*_eff_ was ca. 9–10% larger than its starting value. The apparent diffusion enhancement toward the end of reaction can be explained considering the larger size of copper triazolide (species **6**) and especially copper metallacycle (species **5**) compared to the product triazole molecule.

Finally, we monitored the time dependence of the *D*_eff_ of the catalyst ascorbate molecule using the well-resolved signal #2 (SI, Figure S3). Unlike the reactant alkyne and azide and product triazole molecules, the starting *D*_eff_ of ascorbate molecule was slightly (ca. 2%) larger than the reference *D*_0_ of 5.8 × 10^–10^ m^2^·s^–1^; however, with the progress of the reaction, its *D*_eff_ slowly returned to the reference value. The initial increase in the mobility of the ascorbate molecule is probably caused by the small increase in the temperature and the resultant decrease in sample viscosity. It may also be caused, at least partially, by a signal overlap between ascorbate and its faster diffusing oxidation products generated after addition of Cu(II) ions. It is also notable that no considerable change in the diffusion of solvent molecules was detected.

**Figure 5:**
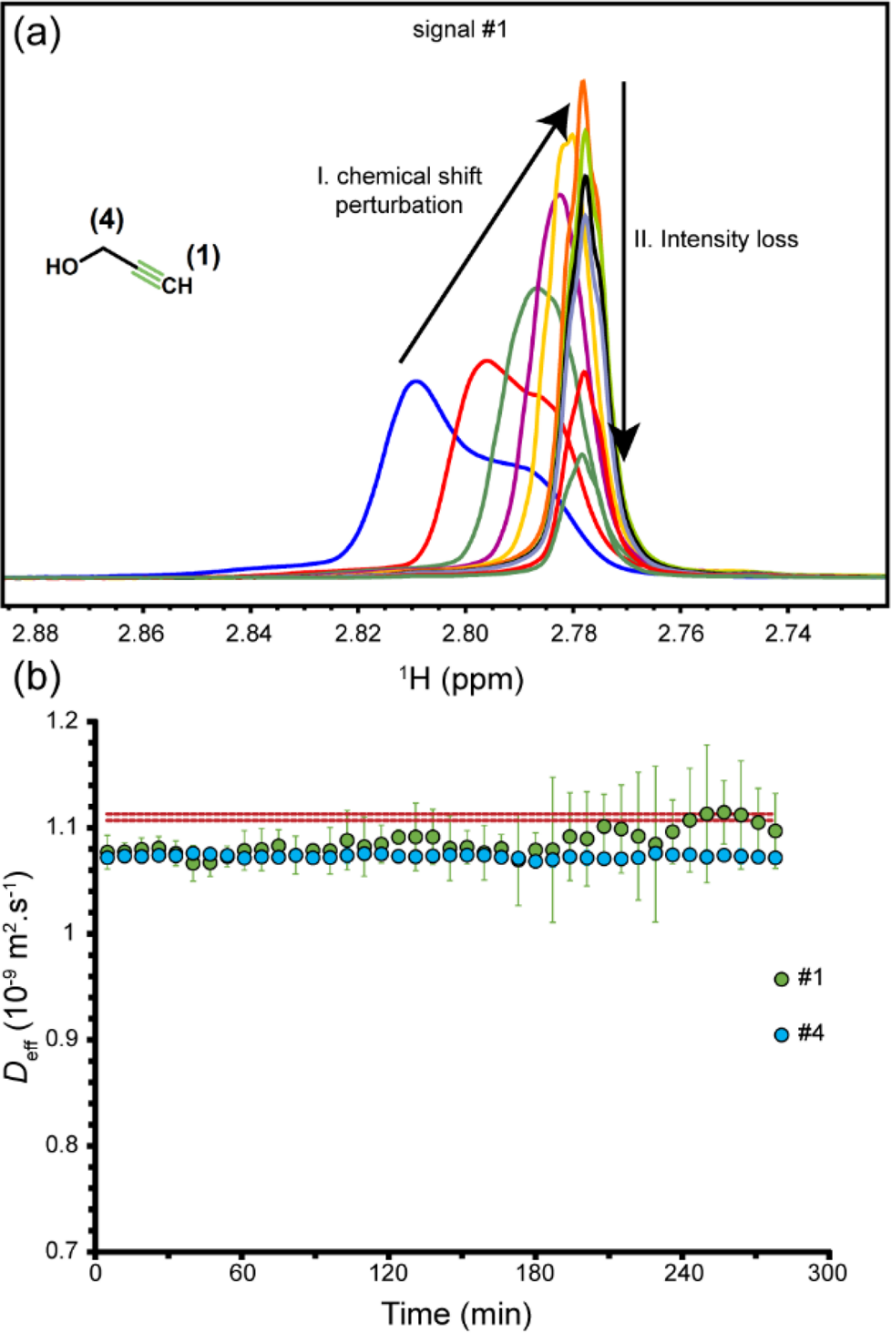
Cu(I) coordination of alkyne in the absence of azide. In (a), time-dependent changes in the chemical shift and intensity of signal #1, belonging to the terminal proton, are shown. As shown in (b), the effective diffusion coefficient of alkyne (*D*_eff_) determined through its signals #1 and #4 is smaller than the reference *D*_0_ value (average ± std dev, dashed line) and remains nearly constant during the coordination process.

To investigate whether the mobility alterations of alkyne are caused by copper π-coordination and/or σ-bond formation alone or further progression into reaction cycle underlies it, we studied a mixture of alkyne and catalyst (copper sulfate and sodium ascorbate), as in the original reaction mixture, but without azide. Consequent to the absence of azide, the reaction cycle would be stopped at step II, where 2Cu-alkyne complex is formed. As shown in Figure 5, signal #1 underwent a gradual displacement toward upfield chemical shifts along with narrowing of the signal, which was later followed by intensity loss. Interestingly, the upfield direction of signal displacement was opposite to the downfield displacement observed during the full reaction (Figure 2a), indicating that the chemical species underlying signal #1 in the dissected and full reactions were different. Signal #4, however, exhibited time-dependent chemical shift changes toward downfield, similar to those observed in the full reaction. The *D*_eff_ values associated with signals #1 and #4 were ca. 3% lower than the reference *D*_0_ of alkyne; however, in contrast with the full reaction, they remained nearly constant along the reaction and did not show further drop. Taken together, our data point to the presence of multiple alkyne–copper species in rapid equilibrium with each other and propose that an intermediate species other than copper alkyne complexes makes a significant contribution to alkyne mobility decay during the click reaction.

**Figure 6:**
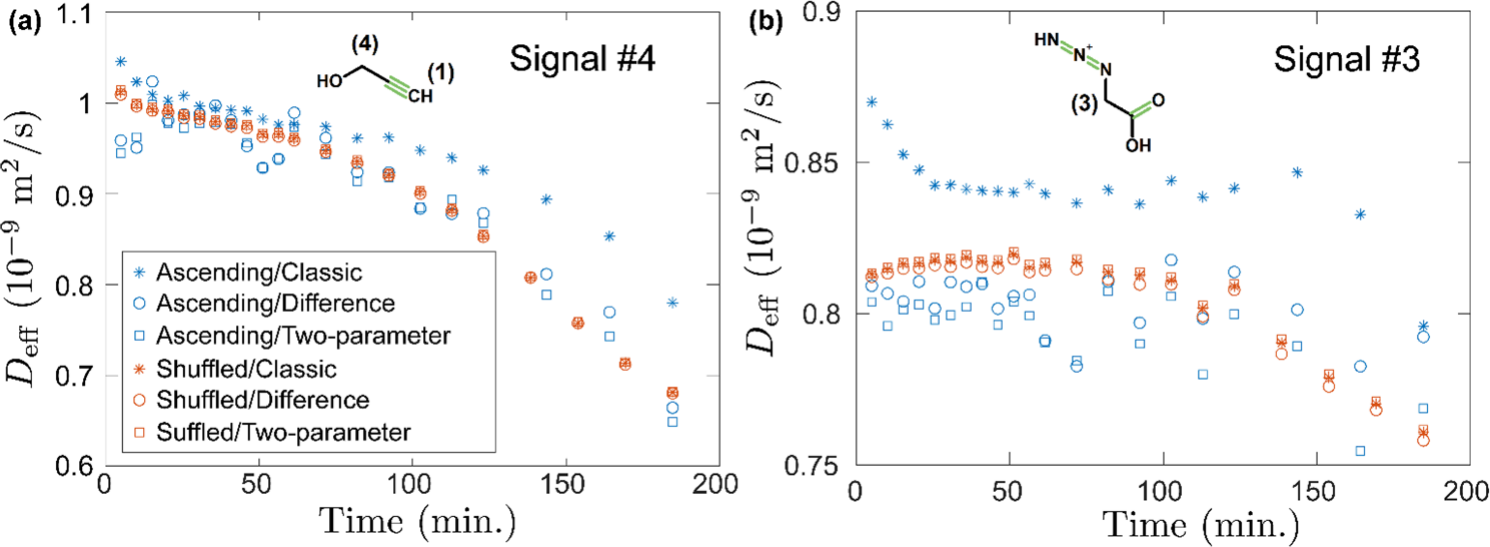
Comparison of PFG-NMR diffusion measurements performed using ascending (standard) and shuffled gradients, each of them analyzed using the “classic ST” equation (see eq S1 in the SI), as well as the two modified ST equations (“difference” and “two-parameter”) described in the main text (see eqs 1 and 2). The signals correspond to the reactants alkyne (a) and azide (b). Notice how, when shuffled gradients are used (red symbols), all three ST equations give identical results. However, when ascending gradients are used (blue symbols), the classic ST equation overestimates the diffusion coefficient relative to the two modified ST equations, which in turn agree with each other and with the shuffled gradient data. The 95% confidence intervals for all data in (a) and (b) are shown in the SI, Figures S4 and S5, respectively.

To verify the importance of using shuffled gradients in the presence of signal intensity changes, we also performed experiments in which gradients were ordered in increasing strength, as in the standard NMR diffusion experiments. The diffusion coefficients obtained from a fit of the signal intensity vs gradient field strength data to the standard ST equation showed significant departures from those obtained using shuffled gradients. One may thus wonder whether it is possible to detect, and potentially correct for, the presence of the systematic artifacts due to signal intensity changes in an experiment that uses increasing gradient strengths. To this end, we devised two modified ST equations that account for the time dependence of signal intensities via two independent methods, which we call the “difference” and “two-parameter” methods. In the difference method, we keep track of the order (*n* = 1, 2, 3, ...) in which the gradients *Q*_*n*_ are applied, and consider only the difference between consecutive gradients, resulting in the modified ST equation,
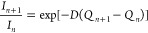
1which will not suffer from artifacts as long as that the signal intensity changes are negligible during the short interval between two consecutive gradient strengths, rather than during the whole measurement as with the standard ST equation. In the two-parameter method, we use a first-order Taylor, i.e., linear, approximation of kinetics for the signal intensity changes during a full measurement starting at time *t*, so that *I*_0_(*t* + Δ*t*) ≈ *I*_0_(*t*) + *I*_0_^′^(*t*)Δ*t* ≡ *I*_0,*t*_ + *I*_0,*t*_^′^Δ*t*, and thus consider the modified ST equation,
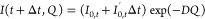
2which, besides the diffusion coefficient, gives information on the average rate *I*_0,*t*_^′^ of signal intensity changes during the measurement. The linear approximation of the reaction kinetics is justified, considering the short duration of the NMR diffusion experiment compared to the time scale of reaction kinetics. Our results confirm that it is indeed possible to detect and correct for such artifacts (Figure 6 and SI, Figures S4 and S5). Whereas the standard ST and the two modified ST equations produced compatible results (in the sense of having overlapping 95% confidence intervals) when applied to the shuffled-gradient data, the results of the standard ST and the two modified ST equations for the increasing-gradient data were incompatible with each other, particularly in the early stages of the experiment (first few minutes for signal #4 and first ∼100 min for signal #3). On the other hand, the two modified ST equations gave results compatible with each other and with those obtained from the shuffled-gradients data.

## Conclusion

Taken together, our results do not show evidence of boosted or active diffusion in the context of the click reaction. The observed diffusion coefficients for the reactants and product of the studied reaction are all smaller during the reaction when compared to theirs in the free form. The changes in the measured diffusion coefficients over the course of the reaction for the NMR signals associated with reactants and products can be explained by the presence of relatively large intermediate species within the reaction cycle with lower diffusivities than both the reactants and the product molecules. The slight transient increase in diffusion observed for the catalyst can be explained as arising from changes in sample viscosity associated with a small temperature increase in the initial stages of the reaction. Moreover, we showed that is possible to detect and correct artifacts arising from signal intensity changes during a diffusion NMR experiment, even *a posteriori*, without the use of shuffled gradients.

The conclusions reached here for the molecular-scale click reaction mirror those that have been reached for a number of nanoscale catalytic enzymes, for which an initial claim of active enhanced diffusion was later shown to be a consequence of passive mechanisms such as conformational changes or subunit dissociation induced by substrate binding and/or catalysis, or even measurement artifacts.^16,18,19,44,45^ From a theoretical perspective, it is important to bear in mind that the momentum imparted by any sudden impulse is dissipated almost instantaneously into the surrounding viscous medium and that any directed motion is randomized quickly by rotational Brownian motion.^15,17−19^

Nevertheless, even in cases where the mechanism behind the diffusion changes is a passive one, unexpected effects can still arise in an out-of-equilibrium setting. For example, diffusion changes due to conformational changes or dissociation can cause directed motion and inhomogeneous steady states in the presence of gradients, and dissociating enzymes may reach and react faster with distant catalytic targets.^46,47^ In this regard, it is also worth noting that during a chemical reaction (even one maintained in a steady state) the populations of reaction intermediates, and therefore the effective diffusion coefficient of the participating molecules, are no longer bound to the constraints of thermodynamic equilibrium and may be influenced by, e.g., the relative kinetic rates of different reactions in the cycle, rather than by free energy differences only. Accounting for the different diffusivities of the various reaction intermediates will thus be important in any setting in which chemical reactions occur inhomogeneously in space, as is certainly the case in biological cells.

## Supporting Information Available

Description of materials, click reaction, and NMR experiments and Figures S1–S5 (PDF)

## Abbreviations

PFG-NMR: pulse field gradient nuclear magnetic resonance
*D*_eff_, *D*_0_: effective and reference diffusion coefficients
CuAAC: Cu(I)-catalyzed azide–alkyne cycloaddition
DFT: density functional theory
