# Supplementary material for: Molecular Diffusivity of Click Reaction Components: The Diffusion Enhancement Question
Source: J Am Chem Soc. 2022 Jan 14;144(3):1380–8. doi: 10.1021/jacs.1c11754 (PMC8796239; doi:10.1021/jacs.1c11754)
Supplement: Supplementary file 1 — ja1c11754_si_001.pdf [file ja1c11754_si_001.pdf]

Molecular Diffusivity of Click Reaction Components: The Diffusion Enhancement Question

Nasrollah Rezaei-Ghaleh[†,#,*], Jaime Agudo-Canalejo[‡], Christian Griesinger[†], Ramin Golestanian[‡,ǁ,*]

[†]Department of NMR-based Structural Biology, Max Planck Institute for Biophysical Chemistry, Am Faßberg 11, D-37077 Göttingen, Germany.

[#]Institut für Physikalische Biologie, Heinrich-Heine-Universität Düsseldorf, Universitätsstr. 1, D-40225 Düsseldorf, Germany.

[‡]Department of Living Matter Physics, Max Planck Institute for Dynamics and Self-Organization, Am Faßberg 11, D-37077 Göttingen, Germany.

[ǁ]Rudolf Peierls Centre for Theoretical Physics, University of Oxford, Oxford OX1 3PU, United Kingdom

Experimental Section

Materials

Propyl-2-ny-ol (propargyl alcohol), sodium ascorbate, copper(II) sulfate pentahydrate and trimethylsilane (TMS) were from Sigma-Aldrich and 2-azido-aceticacid from Jena Bioscience. Deuterated solvent (>99.95% D_2O) was from Cambridge Isotope Laboratories.

CuCAA click reaction

The Cu(I)-catalyzed Alkyne-Azide Cycloaddition (CuAAC) click reaction was triggered by adding 16 mM copper sulfate and 64 mM sodium ascorbate to a mixture of 200 mM propyl-2-ny-ol (henceforth, alkyne) and 200 mM 2-Azido acetic acid in D_2O.[1] The final volume of reaction mixture was 160 μL. The reaction mixture was then immediately transferred to NMR tubes for measurements. The stock solution of sodium ascorbate was freshly prepared each time, in order to minimize its oxidative degradation.

NMR experiments

NMR measurements were performed on an 800 MHz Bruker (Germany) spectrometer, equipped with a cryogenic TCI probe with a z-axis gradient coil. The standard NMR tubes (hilgenberg, Germany) with inner (ID) and outer diameters (OD) of respectively 2.36 and 2.95 mm were used for NMR measurements. The NMR experiments were performed at 298 K, for which the temperature was controlled to ±0.05 K using the Bruker VT unit calibrated through a standardized thermocouple. The temperature stability was checked using the thermocouple before and after our NMR experiments and further verified by measurement of a standard NMR sample containing 99.8% methanol-d4 (NMR thermometer) for 4 hours, the typical duration of the studied click reaction system. Through a multi-slice spatially encoded measurement of the NMR thermometer sample the temperature gradient along the z-axis of the NMR sample was shown to be negligible.[2] The NMR samples contained 100% D_2O, in which the deuteron NMR signal was used for frequency locking. Whenever needed, a small amount of TMS was added into NMR sample and its proton signal was used for chemical shift referencing (0.000 ppm).

The kinetics of click reaction were monitored through standard real-time 1D ^{1}H pulse-acquire experiments, in which the first NMR experiment was started shortly (3-5 minutes) after addition of catalysts, i.e. copper sulfate and ascorbate sodium, to reaction mixture. To ensure an almost complete recovery of longitudinal magnetization for different protons (of reactants, catalysts, intermediates and product), a relatively long recycle delay of 10 s was used in kinetic experiments. However, considering the changes induced in spin-lattice relaxation times (T_1) of all the signals, as shown in [3] and mainly due to changes in the concentration of paramagnetic Cu(II) ions along the reaction, we cannot exclude the possibility of incomplete magnetization recovery, especially at the later stages of the click reaction and/or for protons undergoing exchange with solvent. The average temperature of NMR samples during click reaction was determined using the known temperature-dependence of the chemical shift of residual HDO proton signal (referenced with TMS proton signal, see above).[4] It should however be noted that the possible pH variation along the click reaction induced by deprotonation/reprotonation at steps **II** and **VI** of the click reaction (scheme 1 in the main text) may have partially contributed to water proton chemical shifts. Since no reversal of water proton chemical shifts were observed during the click reaction, the contribution of pH variation to water proton chemical shifts seems to be negligible.

Pulse field gradient (PFG)-NMR diffusion experiments were measured using the standard or modified "stimulated echo bipolar gradient pulse pairs with one coil" (stebpgp1s) or the convection-compensating "double-stimulated echo bipolar gradient pulse pairs with three coils" (dstebpgp3s) sequences.[5] The diffusion delay (big delta, Δ) of 25 ms and diffusion gradient length (little delta, δ) of 2.25 ms were used for diffusion experiments. These values were chosen after exploring the parameter space (big and little deltas ranging over 25-100 ms and 2.25-3.5 ms, respectively) and showing that at the chosen diffusion times the diffusion coefficients obtained through standard and convection-compensating sequences were effectively identical. In this regard, it should be noted that it is crucially important to avoid potential biases introduced by reaction-induced convection, as it would otherwise lead to overestimation of the measured diffusion coefficients, in particular for molecules with relatively small diffusion coefficients (e.g. in the case of click reaction, the relatively large intermediate species are expected to be affected more than the smaller reactant and product molecules).

To account for the time-dependent changes in NMR signal intensities due to click reaction (reactant consumption, product formation) during PFG-NMR experiment, the stebpgp1s and dstebpgp3s sequences were modified to allow the application of gradient field strengths in a random "shuffled" order, and shorten the time interval between different gradient strengths through single-scan-interleaved acquisition scheme. For the sake of comparison, the PFG-NMR experiments were also conducted in the standard manner, i.e. with ascending gradients ramping linearly from 5 to 95% or descending gradients ramping linearly from 95 to 5%. The gradient strengths (in %, relative to maximum gradient strength) were as follows: ascending gradients scheme (0.050000, 0.131818, 0.213636, 0.295455, 0.377273, 0.459091, 0.540909, 0.622727, 0.704545, 0.786364, 0.868182, 0.950000), descending gradients scheme (0.950000, 0.868182, 0.786364, 0.704545, 0.622727, 0.540909, 0.459091, 0.377273, 0.295455, 0.213636, 0.131818, 0.050000) and shuffled gradients scheme (0.050000, 0.950000, 0.459091, 0.868182, 0.377273, 0.131818, 0.540909, 0.213636, 0.786364, 0.295455, 0.704545, 0.622727). A gradient recovery delay of 200 μs and an eddy current delay of 5 ms were used. The number of scans were 8 and 16, respectively for stebpgp1s and convection-compensating dstebpgp3s NMR pulse sequences, with a total recycle delay (acq+d_1) of 3 s and duration of each PFG-NMR experiment of 6 and 10 minutes.

To obtain the diffusion coefficients (D), the gradient-dependent intensity attenuation of NMR signals were fitted to Stejskal-Tanner (ST) equation,

$$I = I_0 \exp(-DQ) \quad \text{(eq. S1)}$$

with $Q = \gamma^2\delta^2\left(\Delta - \frac{\delta}{3}\right)g^2$, where γ is gyromagnetic ratio of proton, g is gradient strength and little and big delta (δ, Δ) are as defined above. The diffusion coefficients obtained from linear or non-linear fitting were identical within 1% margin of error. The fitted data are presented as three-point averages, where each point is weighted (inversely) by the sum of squared errors (SSE) of the fits. The magnetic field gradient was calibrated using a 99.95% D_2O sample and the known diffusion coefficient of HDO molecules at 25 °C ($1.900 \pm 0.004 * 10^{-10}$ $m^2.s^{-1}$).[6] To account for the time-dependence of reactants and product signal intensities during the catalyzed reaction, the NMR diffusion data were also analyzed using two modified versions of ST equations (see the main text).

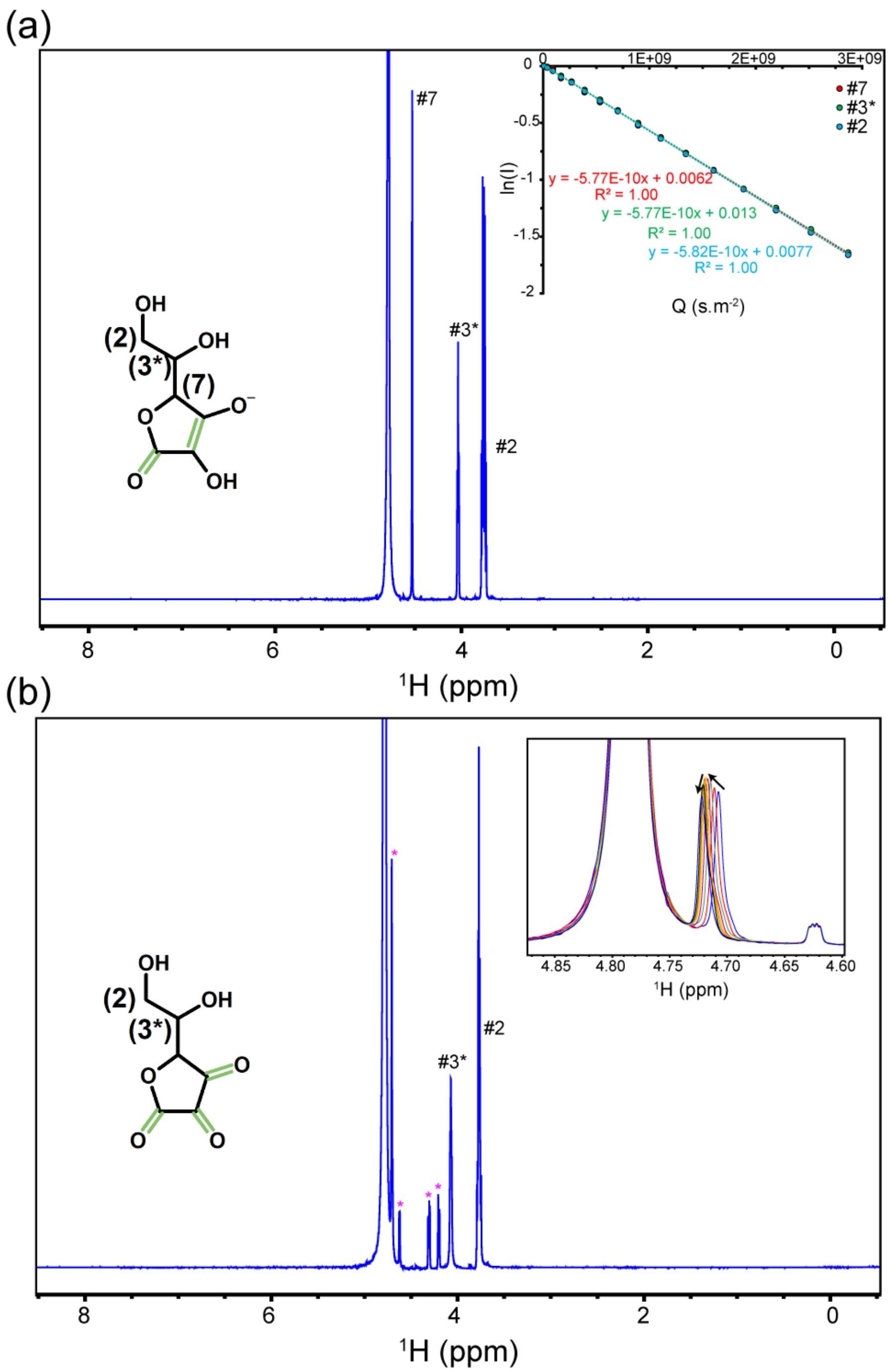

Figure S1. 1D ^{1}H NMR spectra of the catalyst of click reaction (sodium ascorbate, 64 mM), measured in the absence (a) or presence of 16 mM CuSO4 (b). Upon addition of $CuSO_4$, the signal 7 disappears and four new signals, probably belonging to the oxidation products such as dehydroascorbic acid, emerge (marked with pink asterisks). The NMR signals are assigned according to the shown 2D chemical structures of ascorbate and dehydroascorbic acid. In (a), the Inset shows the gradient-dependent NMR intensity attenuation in log-quadratic scale for three signals of ascorbate molecule, where the linear slopes represent the reference diffusion coefficient (D_0) of ascorbate molecule. The Inset of (b) shows time-dependent changes in one of the newly emerged peaks following $CuSO_4$ addition.

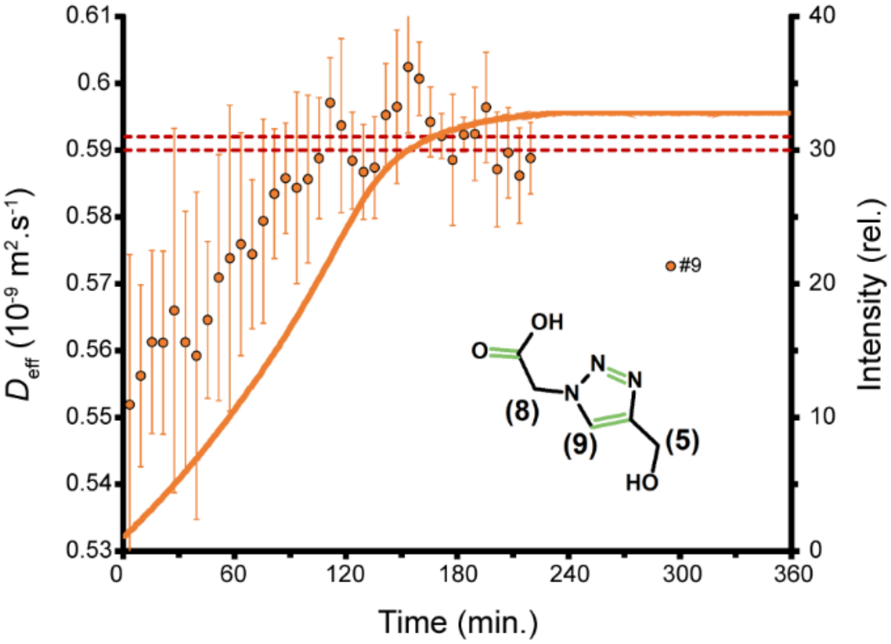

Figure S2. Diffusion of the product molecule during click reaction monitored through real-time PFG-NMR experiments. The effective diffusion coefficient (D_{eff}) of product molecule (triazole) determined via its NMR signal 9 exhibits gradual rise over the course of click reaction (see also Fig. 4c). The reference diffusion coefficient (D_0, average ± stdev) of triazole molecule is shown as dashed lines. The kinetics of click reaction is represented by changes in the intensity of signal 9 (solid line).

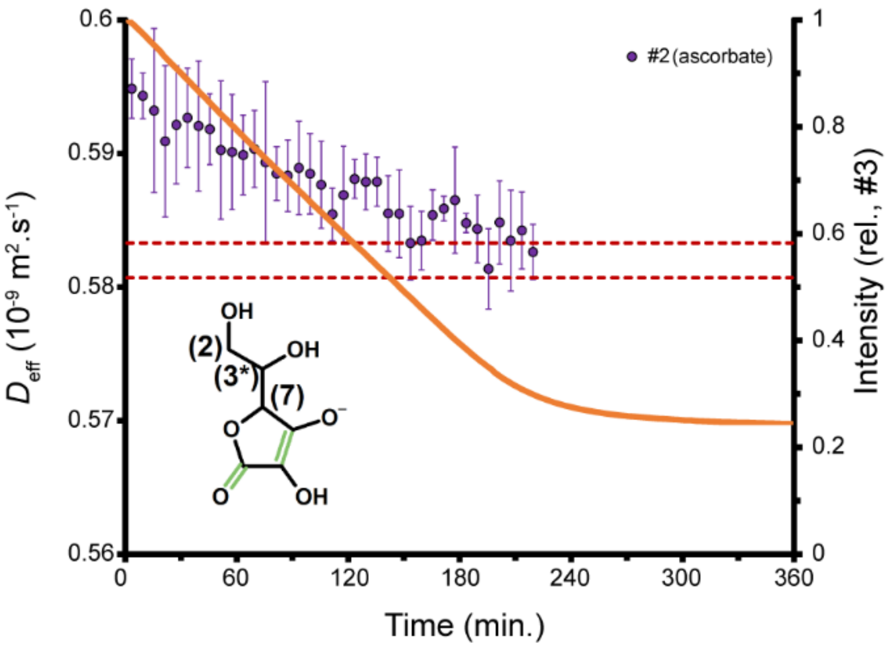

Figure S3. Diffusion of the catalyst ascorbate molecule during click reaction monitored through real-time PFG-NMR experiments. In the beginning of click reaction, the effective diffusion coefficient (D_{eff}) of ascorbate is slightly (ca. 2%) larger than the reference diffusion coefficient (D_0, average ± stdev shown as dashed lines), but slowly decays towards the reference value along with the progression of click reaction. The kinetics of click reaction is represented by changes in the intensity of signal 3 belonging to the reactant azide molecule (solid line).

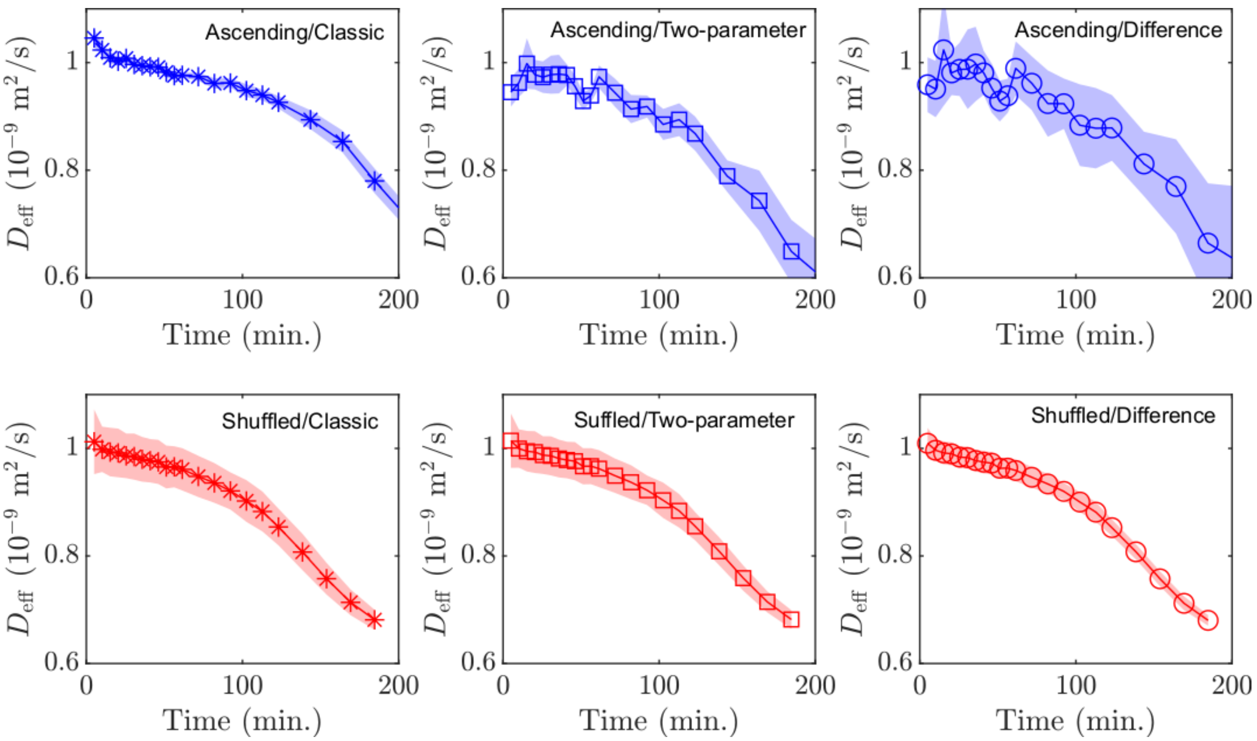

Figure S4. Comparison of PFG-NMR diffusion measurements and analysis methods for the reactant alkyne (Signal #4), corresponding to the same six series shown in Fig. 6(a) of the main text, but here including 95% confidence intervals (shaded areas). Data was collected using ascending (top row) and shuffled (bottom row) gradients, and each was analyzed using the "classic ST" equation, see eq. (S1), as well as the two modified ST equations ("difference" and "two-parameter") described in the main text, see eqs. (1) and (2). When shuffled gradients are used, all three analysis methods give compatible results. When ascending gradients are used, however, the results of the classic ST analysis are incompatible with those obtained from the two modified ST analyses (which are compatible with each other and with the shuffled gradient results). For this signal, the incompatibility is most apparent in the early stages of the reaction (first two datapoints), but it is also present in the later stages of the reaction when one compares the classic and two-parameter analyses.

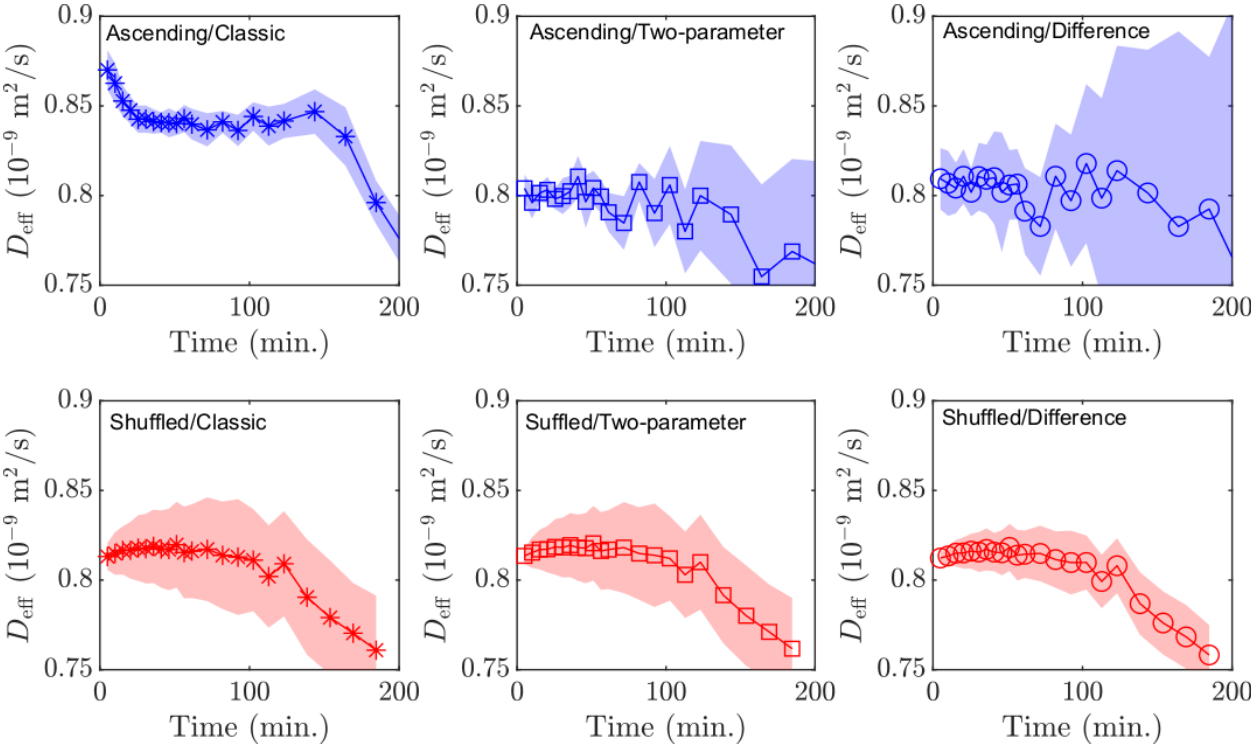

Figure S5. Comparison of PFG-NMR diffusion measurements and analysis methods for the reactant azide (Signal #3), corresponding to the same six series shown in Fig. 6(b) of the main text, but here including 95% confidence intervals (shaded areas). Data was collected using ascending (top row) and shuffled (bottom row) gradients, and each was analyzed using the "classic ST" equation, see eq. (S1), as well as the two modified ST equations ("difference" and "two-parameter") described in the main text, see eqs. (1) and (2). When shuffled gradients are used, all three analysis methods give compatible results. When ascending gradients are used, however, the results of the classic ST analysis are incompatible with those obtained from the two modified ST analyses (which are compatible with each other and with the shuffled gradient results). For this signal, the incompatibility is most apparent in the early stages of the reaction (first 100 min.).

Supplementary References

1. Worrell, B. T.; Malik, J. A.; Fokin, V. V., Direct Evidence of a Dinuclear Copper Intermediate in Cu(I)-Catalyzed Azide-Alkyne Cycloadditions. *Science* **2013,** *340*, 457-460.
2. Castanar, L.; Nolis, P.; Virgili, A.; Parella, T., Simultaneous multi-slice excitation in spatially encoded NMR experiments. *Chem. Eur. J.* **2013,** *19*, 15472-5.
3. Gunther, J. P.; Fillbrook, L. L.; MacDonald, T. S. C.; Majer, G.; Price, W. S.; Fischer, P.; Beves, J. E., Comment on "Boosted molecular mobility during common chemical reactions". *Science* **2021,** *371*, eabe8322..
4. Webb, A. G., In *Annual Reports on NMR Spectroscopy*, Academic Press: 2002; Vol. 45, pp 1-67.
5. Swan, I.; Reid, M.; Howe, P. W. A.; Connell, M. A.; Nilsson, M.; Moore, M. A.; Morris, G. A., Sample convection in liquid-state NMR: Why it is always with us, and what we can do about it. *J. Magn. Reson.* **2015,** *252*, 120-129.
6. Mills, R., Self-Diffusion in Normal and Heavy-Water in Range 1-45 Degrees. *J. Phys. Chem.-Us* **1973,** *77*, 685-688.
